# Protection Applied to the Practice of Medicine

**Published:** 1890-01

**Authors:** 


### PROTECTION APPLIED TO THE PRACTICE OF MEDICINE.

The following editorial with the above title in the New York *Medical Journal*, for December 14, 1889, calls attention to an evil that we consider of sufficient importance to reproduce entire what our able contemporary says on the subject. We bespeak for it a careful reading :

The very large number of Americans who winter in the south of Europe, whether for purposes of recreation or for the recovery of lost health, will hear with astonishment and dismay of the recent action of the French Government concerning English physicians practising abroad. Formerly a foreign physician wishing to practise in the Riviera could qualify by reporting himself at Marseilles and passing the by no means insignificant examination for the grade of *officier de santé*. This was a considerable obstacle and deterred many from pitching their tents in the French winter resorts. But it seems that even this protection is not sufficient. At Nice, Cannes, and Mentone —places, be it remembered, built up entirely by the recommendations of English physicians—the whole of the English and American practice (and very good practice it is in the season) has been in the hands of the well-established English-speaking physicians settled there. This state of affairs seems to have caused a great deal of jealousy among the native doctors, who, as elsewhere on the continent, regard Anglo-American travel as an institution specially devised by Providence for their maintenance. Pressure has been brought to bear upon the French Government, by the representatives of these persons, of such weight as to force M. Fallières, the Minister of Public Instruction, to inform the English ambassador in Paris, with respect to English medical men who may be seeking authority from the French Government to practise in France, that it will not be possible in future for his Department to give the same favorable consideration to applications of this kind as has hitherto been accorded them, whether received directly by the Minister or through the British Embassy, and that the applications will be refused unless "in instances presenting very exceptional claims."

The meaning of this pronunciamento is plainly to the effect that after date no English doctor is to practise in France. Those who have already gained a high professional position in the cities and towns which Englishmen and Americans most frequent will, it is presumed, not be expelled; but they are not immortal, and a few years will see the race die out, and then the French doctor will have it all his own way; he alone will have the honor and glory of looking at the tongue and feeling the pulse of John Bull and Brother Jonathan.

We regard this as a very serious matter, requiring joint action on the part of our Government and that of Great Britain. When we consider the vast number of our countrymen who spend their time in France, it seems outrageous that they should practically be denied the privilege of obtaining necessary advice, to say nothing of sympathy and consolation, from one speaking their own tongue. A foreign doctor in no way takes the place of one accustomed to our ways of thought and action, even if he does speak English or even if we do speak his language. The French law, if carried out, will render it a punishable offense for a young American or English doctor to travel with a patient in France, and a New York or London consultant will not find in foreign cities a physician to whom he can transfer a patient whose case requires careful explanation.

If the French Government would but inquire into the causes of the influx of visitors to these winter resorts, it would find that the Anglo-American immigration originated largely in the recommendations of those speaking our language. Smollett, an English doctor, brought Nice to public notice. What would Cannes, with its sixty hotels and its floating population of ten thousand, be to-day were it not for the notice into which it was brought by Lord Brougham, and for the recommendations of such authorities in chest disease as Williams and Walshe? As for Mentone, it was made by Bennett. And now, having enjoyed for so many years the products of the conversion of the English sovereign and the American dollar into the French franc, the Department of Public Instruction of the Republic is about to perform that old operation described in detail by Æsop and known to the public as killing the goose that lays the golden eggs.

